# Biochemical evidence of cell starvation in diabetic hemodialysis patients

**DOI:** 10.1371/journal.pone.0204406

**Published:** 2018-09-27

**Authors:** Masako Fujiwara, Itiro Ando, Keisuke Satoh, You Shishido, Kazuhito Totsune, Hiroshi Sato, Yutaka Imai

**Affiliations:** 1 Department of Planning for Drug Development and Clinical Evaluation, Graduate School of Pharmaceutical Sciences, Tohoku University, Sendai, Miyagi, Japan; 2 Koujikai Nagamachi Clinic, Sendai, Miyagi, Japan; 3 Midorinosato Clinic, Iwanuma, Miyagi, Japan; 4 Laboratory of Clinical Pharmacology and Therapeutics, Graduate School of Pharmaceutical Sciences, Tohoku University, Sendai, Miyagi, Japan; 5 Tohoku Institute for Management of Blood Pressure, Sendai, Miyagi, Japan; University Medical Center Utrecht, NETHERLANDS

## Abstract

Recently, the ratio of patients with diabetes mellitus (DM) among hemodialysis (HD) patients has increased to become the largest sub-population. Their prognoses are significantly worse than those of patients without diabetes (non-DM). In the present study, 10 DM patients who did not take meals and 10 non-DM patients who took meals during HD sessions were investigated. The time courses of the change in plasma levels of metabolites during HD were determined. DM patients exhibited decreased plasma levels of lactate, pyruvate and alanine and dramatically increased levels of ketone bodies. At the end of HD, the plasma levels of lactate, pyruvate, alanine and ketone body were 0.46 ± 0.07, 0.026 ± 0.01, 0.12 ± 0.04 and 0.26 ± 0.04 mM (mean ± standard error), respectively. The profile was ‘hypolactatemia and hyperketonemia’, indicating non-homeostasis. Glycolysis and tricarboxylic acid cycle were suppressed, and the oxidation of fatty acid was accelerated, indicating starvation, even though high amounts of glucose (150 mg/dl) in dialysate were supplied continuously to the bloodstream. In contrast, the plasma levels of lactate, pyruvate, and alanine in the non-DM patients were increased, with the levels of ketone body remaining low during HD to maintain homeostasis, indicating accelerated glycolysis. Furthermore, their plasma levels of insulin increased from 8.1 ± 1.4 to 19.8 ± 3.4 μU/ml, which indicated endogenous secretion stimulated by glucose in dialysate and meal intake. In contrast, in the DM patients, the levels decreased from 19.2 ± 3.4 to 5.5 ± 1.1 μU/ml. This value was the lower limit of the normal range. The depletion of the insulin through extracorporeal circulation may inhibit the transportation of glucose from the blood into the muscles, with the consequence of cell starvation. Such cell starvation along with lipolysis every two days may accelerate proteolysis and affect the prognosis of DM patients.

## Introduction

The population of hemodialysis (HD) patients is growing globally [1]. Recently, the ratio of patients with diabetes mellitus (DM) has increased to become the largest sub-population of HD patients. Sarcopenia is a severe complication in DM-HD patients (an age-related decline in physical performance or muscle atrophy). This complication is often combined with protein energy wasting, chronic inflammation, infectious disease or cardiovascular disease [2–6]. Consequently, the prognosis is significantly worse than in patients without diabetes (non-DM), and in Japan, the 5-year survival rate is approximately 50%. In contrast, non-DM patients with adequate HD therapy can expect to live for up to around 40 years [7].

In HD therapy, not only uremic toxins but also small metabolites of nutrients and physiologically necessary bioactive molecules, including (but not limited to) amino acids, lactate and pyruvate, are indiscriminately filtered [8]. In our previous study, we demonstrated by proton nuclear magnetic resonance (^1^H NMR) spectroscopy of dialysate that HD resulted in the substantial removal of these metabolites from the blood [9]. Previous studies have reported that 5–8 g of amino acids exit the blood per HD session, causing the malnutrition characteristic of HD patients [10, 11].

However, few studies have investigated the metabolic responses to the removal of metabolites associated with the main energy productive pathway by HD. Our previous study of the metabolic responses of non-DM patients showed glycolysis acceleration with increased levels of lactate, pyruvate and alanine at the middle time point during HD [9]. The present study aimed to investigate the metabolic responses of DM patients during HD using a recently developed high-flux membrane and dialysate with a glucose concentration of 150 mg/dl, which is higher than the physiological blood glucose concentration.

Most previous studies on metabolic alterations in HD patients have examined differences in the plasma levels of metabolites before and after HD [10]. Such methods were unable to show the overall balance of the metabolism, since certain components of metabolites are secreted from the tissue during HD. Furthermore, even if the pre- and post-HD values are equal, it might not indicate an absence of changes during HD. We previously described the merits of the ^1^H NMR technique in the quantification of plasma metabolites using spent dialysate [9, 12, 13]. Our study showed that patterns in the time course of changes in metabolite concentrations during HD were reproducible and characteristic for non-DM patient [9].

In the present study, we showed distinct time courses of metabolite concentrations during HD between non-DM and DM patients. To investigate the energy metabolisms of the two patient groups, plasma immunoreactive insulin (IRI) levels before and after HD were measured.

## Patients and methods

### Patients

The study protocol was approved by the Ethics Committees of both Koujinkai Hospital (Koujinkai Hemodialysis Clinic, Miyagi, Japan) and the Graduate School of Pharmaceutical Sciences in Tohoku University (Miyagi, Japan) and was executed in accordance with the Declaration of Helsinki. Twenty Japanese patients undergoing maintenance HD with three 4-h sessions per week were recruited, and their written informed consent was obtained. Ten patients with type 2 DM and 10 non-DM patients were randomly selected from 90 patients undergoing stable HD during the day in Koujinkai Nagamachi Clinic. The DM patients did not consume lunch, nor did they receive insulin injections or other oral medicines for DM during HD. Outside of the HD sessions, four patients were treated without anti-diabetic medicines, four patients were treated insulin injection, and two received oral diabetic medications (S1 Table).

Their basic characteristics are shown in Table 1. The non-DM patients included nine patients who consumed lunch that had been described in our previous study [9] and one additional patient who also consumed lunch during HD. The common practice in HD clinics seems to be that non-DM patients consume meals during HD, while DM patients do not for the sake of maintaining glycemic and blood pressure. The patients were virtually anuric and received dialysis with high-flux polysulfone membranes. The flow rates of blood and dialysate were 200 and 500 ml/min, respectively. The glucose concentration in dialysate was 150 mg/dl. In each group, 7 and 3 patients used dialysate consisting of bicarbonate buffer with acetate (8 mM) and citrate (0.7 mM), respectively.

**Table 1:** Characteristics of the non-DM and DM patients.

	Non-DM	DM	*P*
n	10	10	
Sex (male)	6	6	
Age (years)	65.8 ± 7.1	72.4 ± 8.2	0.025*
HD duration (years)	20.0 ±11.8	7.97 ± 3.3	0.0015**
	(3–36)	(3–12)	
Glucose (mg/dl)	102.2 ± 14.3	115.6 ± 36.0	0.15
Weight (kg)	52.1 ± 10.2	55.3 ± 9.3	0.23
Creatinine (mg/dl)	10.6 ± 1.62	10.8 ± 1.6	0.3
Albumin (g/dL)	3.73 ± 0.27	3.76 ± 0.15	0.48
Hemoglobin (g/dL)	11.1 ± 0.5	11.8 ± 1.97	0.13
Hematocrit (%)	32.7 ± 2.1	33.7 ± 1.27	0.08
HbA1c (%) (NGSP)	-	6.03 ± 0.54	
Kt/V	1.64 ± 0.28	1.66 ± 0.33	0.44
Dialysate (Ac : Ct)	7 : 3	7 : 3	

### Sample collection and preparation

At 2-month intervals, spent dialysate was collected individually from the 20 patients over a 10-month period (a total of 5 HD sessions). In each session, dialysate was collected at 15 min (initial), 30 min, 1 h, 2 h, 3 h and 4 h (final) after the initiation of HD. For each patient, plasma samples were collected once before and after HD during the study period. Dialysate and plasma samples were stored in standard plastic tubes at -25°C. They were evaluated within one week after collection. Dialysate samples were thawed on ice immediately before use. For NMR measurement, 50 μl of deuterium oxide (to provide a field-frequency lock) and sodium 3-(trimethylsilyl) propionate 2, 2, 3, 3-*d*_4_ (TSP) (as an internal chemical shift and an intensity reference) were added to the 550 μl dialysate sample. The sample was centrifuged for 10 min at 15,000 *g*, and then the supernatant was placed into a 5-mm NMR tube.

### Quantitative NMR spectroscopy

The single-pulse ^1^H NMR spectra were recorded using a 600-MHz NMR spectrometer (ECA; JEOL, Ltd., Tokyo, Japan) with an internal probe temperature of 25°C. The water signal was suppressed by a presaturation pulse sequence, and the flip angle was set to 90°. Sixty-four scans were performed for 64 K data points with a spectrum width of 9 kHz. The repetition times for dialysate were set at 42.4 s, which ensured larger than 5×T_1_ (T_1;_ longitudinal relaxation time) [12, 13]. Free induction decay was processed using the software program ALICE2 for Windows (V.6.0; JEOL, Ltd.).

### Quantification of metabolites and plasma IRI

**Fig 1:**
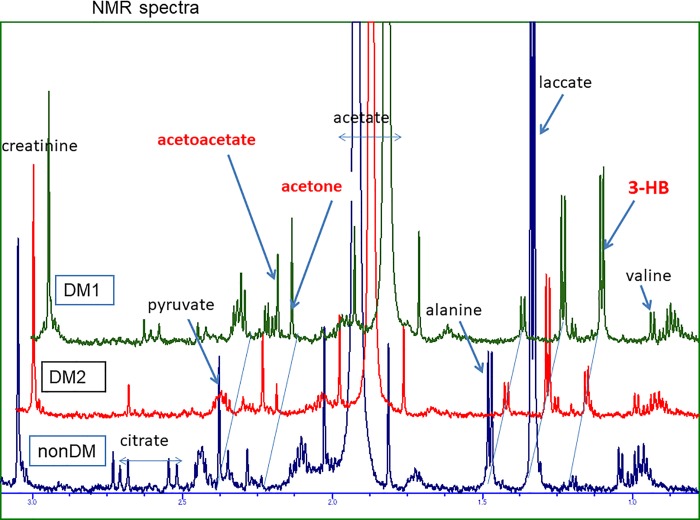
Partial ^1^H NMR spectra of dialysate at 3 h after starting HD from 1 non-DM patient and 2 DM patients. The spectra were aligned where the standard heights were quantitatively determined. This dialysate contained acetate; thus, the acetate peaks were constant and overflowed in the graph.

For the measurement of metabolites in the dialysate, the signals of creatinine (CH_3_, 3.05 ppm), lactate (CH_3_, 1.33 ppm), pyruvate (CH_3_, 2.38 ppm), alanine (CH_3_, 1.47 ppm) and valine (2CH_3_, 0.98, 1.04 ppm) were assigned and quantified based on the spectra (for examples in Fig 1) according to the methods reported in our previous study [9]. Signals other than those noted above, 3-hydroxybutyrate (3-HB) (CH_3,_ 1.20 ppm), acetoacetate (CH_3_, 2.25 ppm), acetone (2CH_3_, 2.22 ppm) and citrate (2half CH_2_, 2.52, 2.69 ppm) were also assigned and quantified.

In our previous study, the signal areas of metabolites (creatinine, valine, alanine and lactate) on ^1^H-NMR spectra of plasma and dialysate were quantified [12]. The conversion ratios of the metabolites levels in dialysate to those in plasma were determined, and their mean value of 3.5 was applied in the present study.

HD patients used two kinds of dialysate. Acetate was quantified using citrate containing dialysate without acetate, and citrate was quantified using acetate containing dialysate without citrate. Both types contained glucose. In the present study, the plasma metabolites that were detected by ^1^H NMR were endogenous and were not contained in the original dialysate.

The plasma levels of IRI were determined using a chemiluminescent immunoassay method (LSI Medience Corp., Tokyo, Japan).

### Statistical analyses

**Fig 2:**
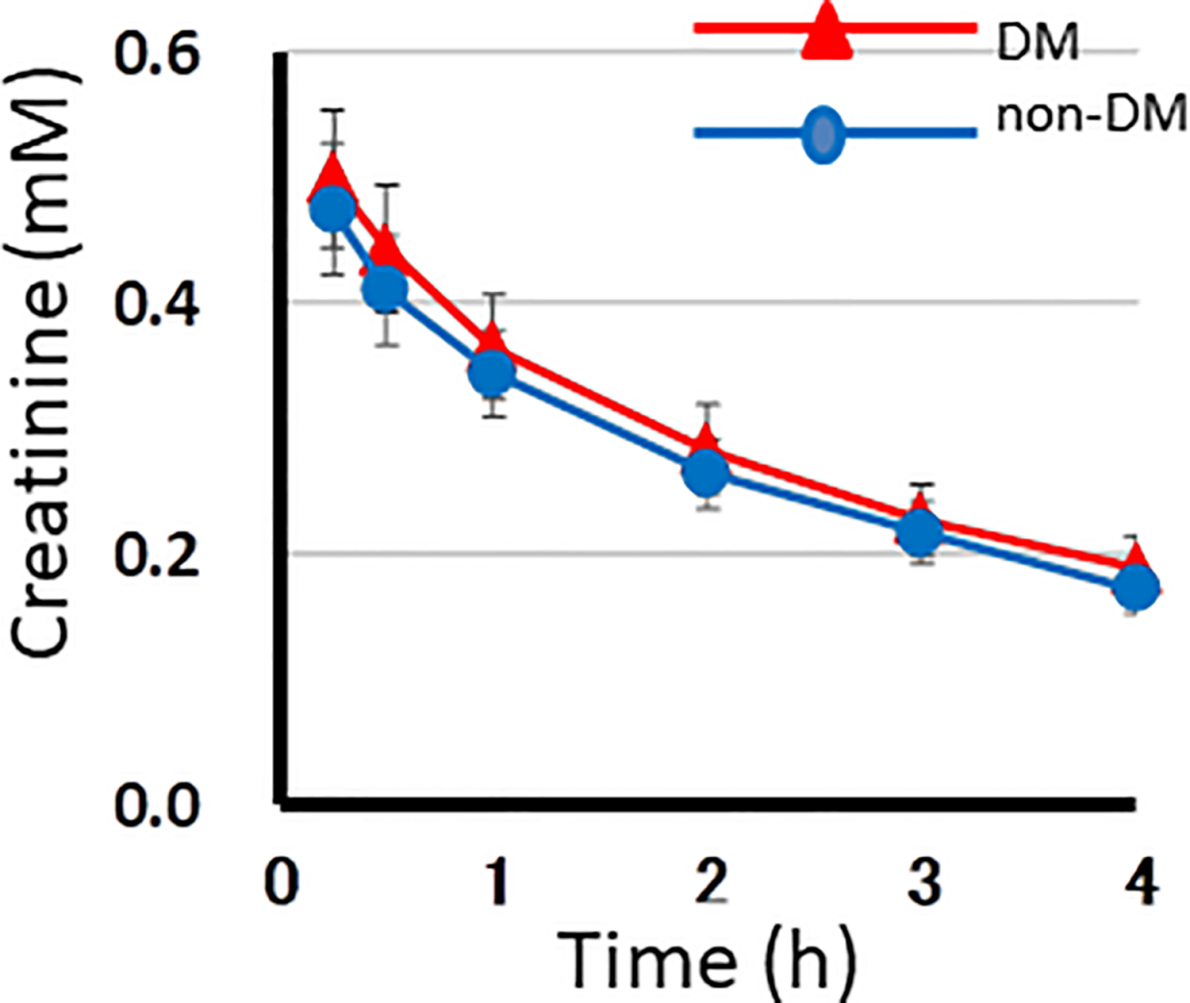
The plasma levels of creatinine during HD in 10 DM and 10 non-DM patients measured by ^1^H NMR spectroscopy. In the graph, the x- and y- axes indicate the hours of the HD session and the plasma levels of creatinine (mM), respectively. Red and blue lines represent for DM and non-DM patients, respectively. Dots and vertical bars indicate the mean and SE of 10 patients in each of 5 sessions, respectively.

**Fig 3:**
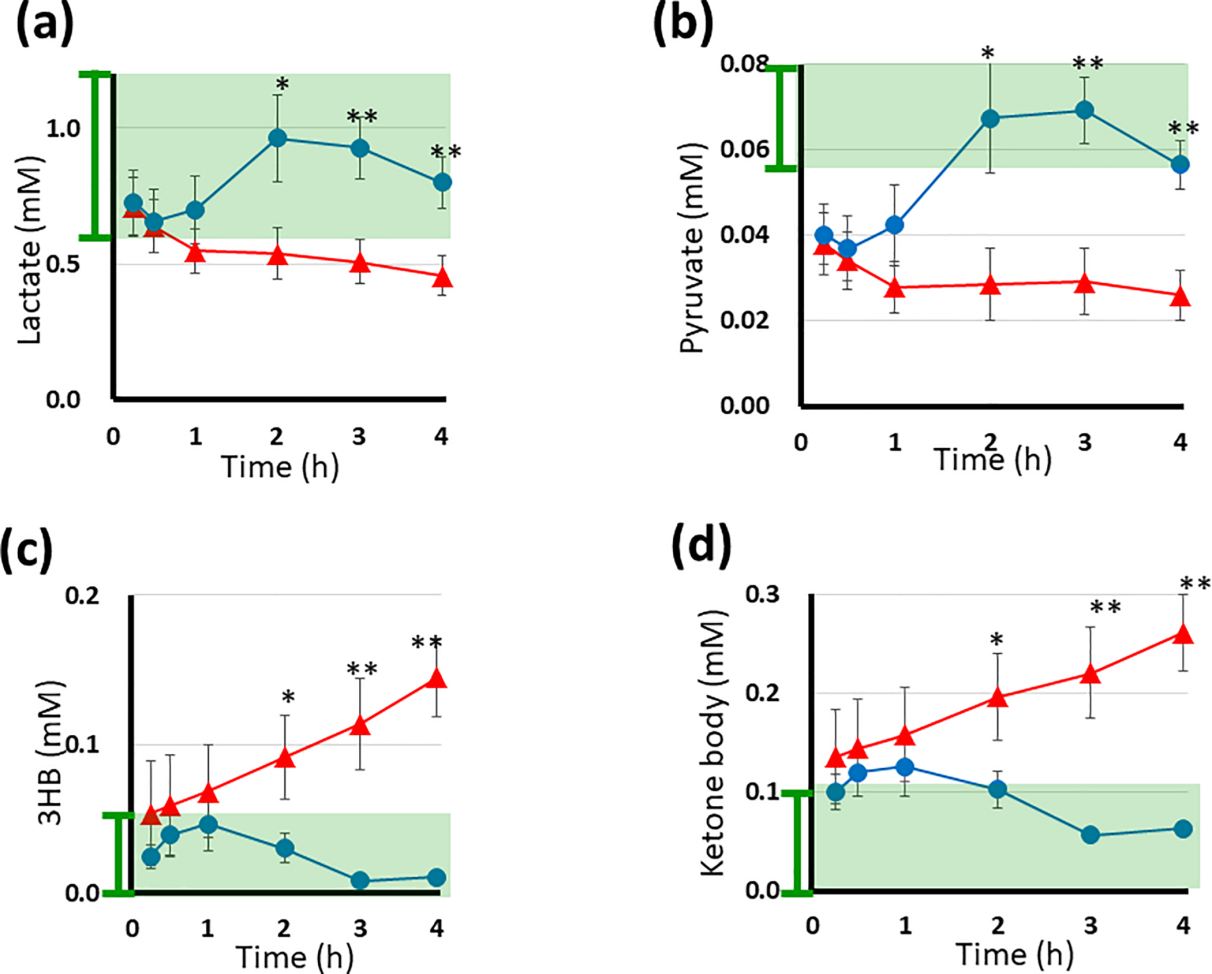
**The plasma levels of lactate (a), pyruvate (b), 3-HB (c) and ketone body (d) during HD in 10 DM and 10 non-DM patients measured by** ^**1**^**H NMR spectroscopy.** In each graph, the x-, y- axes, red, blue lines, and dots, vertical bars represented same as the Fig 2, for lactate, pyruvate, 3-HB and ketone body, respectively. Asterisks above the dots indicate statistical significance at *P* <0.05 (one asterisk) and *P* <0.01 (double asterisks). The ketone body level is indicated as the sum of 3-HB, acetoacetate and acetone. In the graphs, the normal levels are indicated by green shadow boxes with vertical bars to the left of the y-axis.

The biochemical data are presented as the mean ± standard error (SE). The statistical significances in Table 1 and Fig 2 were determined by one-sided Welch’s *t*-tests at the *P* < 0.05 level. Those in Fig 3 were determined by one-sided paired *t*-tests. Analyses were performed using the software program JMP (Ver. 13.0 package; SAS Institute Inc. Cary, NC, USA).

## Results

### Patient characteristics

All 20 patients underwent plasma biochemistry examinations every 2 weeks before HD under usual conditions without fasting. From these data, those measured closest to the time of sample collection for NMR measurement (5 times) were averaged for each patient. The results in 10 DM and in 10 non-DM patients are shown in Table 1. Patients’ characteristics and HD conditions are shown also in Table 1. With the exception of the age and duration of HD, the characteristics of the patients in the two groups were similar. DM patients were older and had a shorter duration of HD than non-DM patients. The SE of the glucose levels in DM patients was larger than that of non-DM patients. The glucose levels of DM patients were not significantly higher than those of non-DM patients, indicating that the glucose levels of DM patients were largely well controlled, which was also reflected by their HbA_1c_ levels.

Table 1 shows the baseline characteristics of the patients with and without DM. The values indicate the mean ± SE or the number (range). HD treatments were performed using bicarbonate dialysis buffer containing 8 mM acetate (indicated as Ac) or 0.7 mM citrate (indicated as Ct). Kt/V is an indicator of the efficacy of HD therapy. An asterisk and double asterisks indicate statistically significant differences, *P* < 0.05 and 0.01, respectively.

### Examples of ^1^H NMR spectra

Fig 1 shows typical examples of the ^1^H NMR spectra in low-frequency (δ 0.75–3.15 ppm) regions of dialysate from 1 non-DM patient and 2 DM patients at 3 h after the initiation of HD. Acetate dialysate was used, and large acetate peaks were observed. The non-DM patient showed large peaks of lactate, alanine and pyruvate along with subtle peaks of ketone bodies (3-HB, acetone and acetoacetate). In contrast, large peaks of ketone bodies and minimal peaks of citrate, lactate and alanine were observed in the two DM patients.

### The time course of the changes in the metabolite concentrations

**Fig 4:**
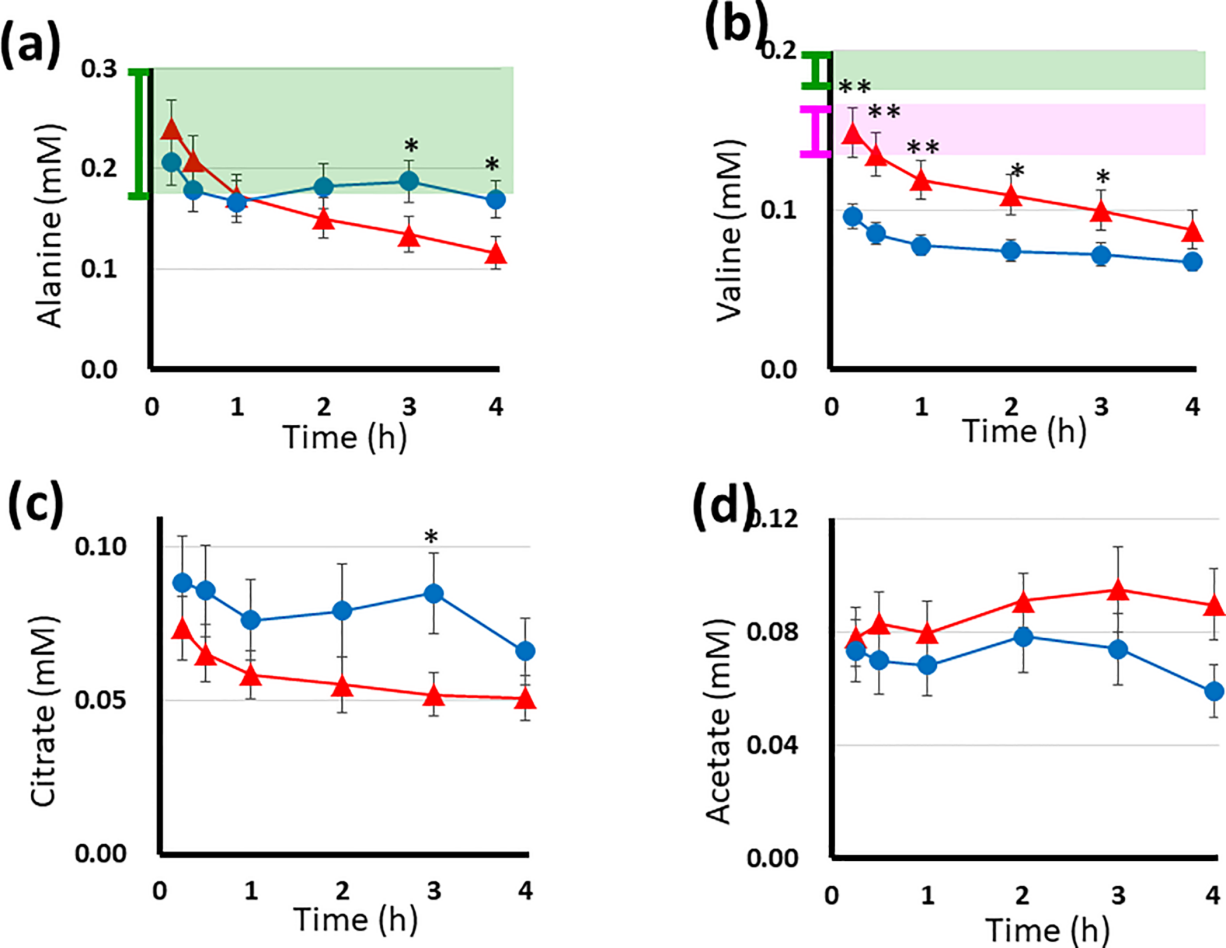
**The plasma levels of alanine (a), valine (b), citrate (c) and acetate (d) during HD in 10 DM and 10 non-DM patients measured by** ^**1**^**H NMR spectroscopy.** In each graph, the x-, y- axes, red, blue lines, and dots, vertical bars represented same as the Fig 2, for alanine, valine, citrate and acetate, respectively. Asterisks above the dots indicate the same as in Fig 3. In the graph (a, b), the green shadow boxes are the same as Fig 3. In graph (b), the standard levels obtained from HD patients are indicated as a pink shadow box with a vertical bar to the left of the y-axis. Graphs (c) and (d) show the citrate levels of seven patients and the acetate levels of three patients in both groups who received dialysate without citrate and without acetate, respectively.

The time course of the changes in plasma metabolite levels during HD in 10 DM and 10 non-DM patients were evaluated by ^1^H NMR spectroscopy of the dialysate. Data from five HD sessions for each patient were averaged. These values were averaged in each group (Figs 2, 3 and 4). The graphs revealed characteristic features of the time course of the changes in the metabolite concentrations in each group.

The creatinine levels (Fig 2) exponentially decreasedsimilarly with the small SE values in the two groups. This indicated that HD was conducted under a stable condition throughout the study [8]. The initial creatinine levels decreased by one-third at the end of HD.

Fig 3A and 3B show the time course of the changes in the plasma levels of lactate and pyruvate. In the non-DM patients, the lactate and pyruvate levels dropped in the initial 30 min, while large increases were observed at 2-3 h after the initiation of HD; the levels then decreased toward the end of the HD session. However, they remained within the normal range throughout HD. In contrast to the non-DM patients, the lactate and pyruvate levels of the DM patients showed a continuous decrease from 0.71 ± 0.11 and 0.46 ± 0.07 mM to 0.04 ± 0.03 mM and 0.026 ± 0.01 mM, respectively. They did not increase during HD. At the end of the HD session, the level of lactate neared the lower limit of the normal range, and the level of pyruvate was less than the normal limit. In each patient group, the lactate and pyruvate levels behaved synchronously. The two metabolites were directly coupled on the metabolic map (see below).

Fig 3C and 3D show the time course of the changes in the concentrations of 3-HB and total ketone bodies. In the non-DM patients, the levels of 3-HB and ketone bodies increased slightly within the first hour, thereafter decreasing and remaining low. These levels were within their normal ranges during HD. In contrast, in DM patients, the initial levels were higher than in non-DM patients (at the higher limit of normal) and showed a large and continuous increase throughout HD. At the end of HD, the level of ketone bodies had increased 2-3-fold, from 0.14 ± 0.05 mM to 0.26 ± 0.04 mM, reflecting slight or mild clinical ketosis. Some DM patients showed very high levels (approximately 1.0 mM) of ketone bodies that exceeded the level of ketosis [14]; one spectrum is shown in the top lane of Fig 1.

Fig 4A shows the time course of changes in the plasma levels of alanine, a typical glycogenic amino acid. In non-DM patients, it decreased within the first hour before increasing slightly around the second and third hours, remaining within the normal range. In contrast, the plasma levels of alanine in DM patients showed a continuous decrease throughout the HD session. Its final level was lower than the normal level (0.18–0.58 mM) [15].

Fig 4B shows the time course of changes in the plasma levels of valine, a branched-chain amino acid. Initially, the valine levels in both groups were below the normal level (0.17–0.32 mM) [16] and gradually decreased further. The initial level of valine in DM patients was within the standard range obtained from HD patients (0.15 ± 0.02 mM) [16]. The valine levels in DM patients were higher than those in non-DM patients during HD.

Fig 4C shows the time course of changes in the citrate levels of seven patients each in the DM and non-DM groups who received dialysate without citrate (representative spectra are shown in Fig 1). In the non-DM patients, an initial decrease in citrate was followed by an increase. Thereafter, citrate levels decreased until the end of HD. In the DM patients, the levels of citrate showed a continuous decrease.

Fig 4D shows the changes in the acetate levels of the three patients who received dialysate without acetate (spectra shown in our previous study [9]). The acetate levels of the DM patients were higher than those of non-DM patients.

### Plasma levels of insulin before and after HD

**Fig 5:**
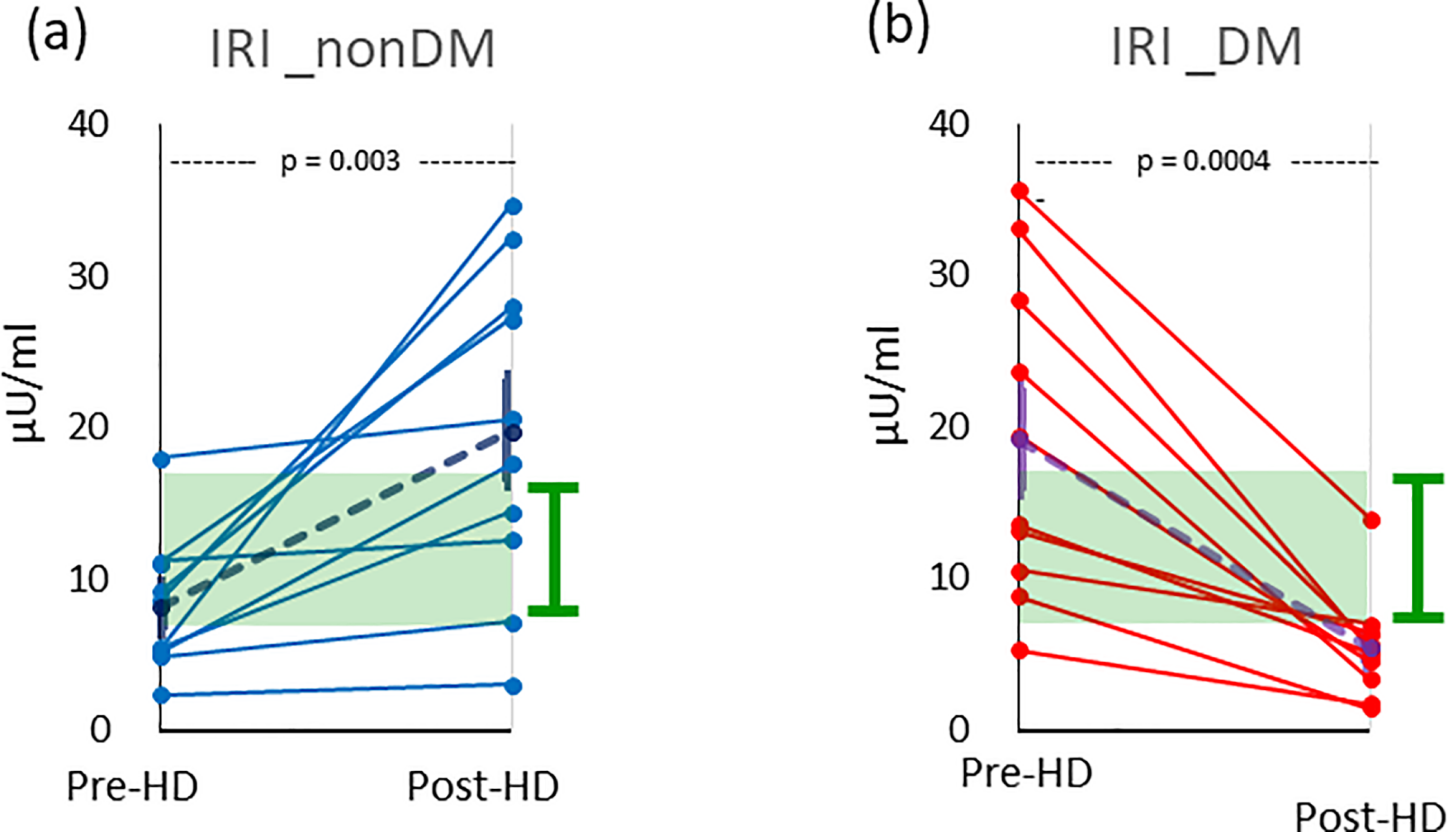
**The plasma insulin levels (**μ**U/ml) before and after HD in 10 non-DM (a) and 10 DM (b) patients, respectively.** The means are depicted as broken lines with SEs. The normal ranges of IRI are indicated as green boxes with vertical bars to the right of the y-axes (a, b).

The plasma insulin (IRI) levels were measured before and after HD. As shown in Fig 5A and 5B, the changes in the IRI levels before and after HD differed significantly between non-DM and DM patients.

In non-DM patients (Fig 5A), the initial IRI levels were 8.1 ± 1.4 μU/ml (mean ± SE), which was near the lower limit of the normal range (5–15 μU/ml) [17], and no decrease in the IRI was observed. In some patients, the values were maintained, while half of non-DM patients showed a marked increase toward the end of the HD session. After HD, the IRI levels in non-DM patients were 19.8 ± 3.4 μU/ml and heterogeneously distributed. A profound increase in the IRI (11.7 ± 3.2 μU/ml) was observed during HD (*P* = 0.003). In contrast, the IRI values of all DM patients decreased after HD (Fig 5B); the initial values were 19.2 ± 3.4 μU/ml and heterogeneously distributed. Most of the DM patients had taken insulin injections in the morning before HD, and their IRI levels dropped rapidly after HD. After HD, the levels were 5.5 ± 1.1 μU/ml, and the distribution was clustered. The decrement in the insulin levels from before to after HD was 13.7 ± 2.8 μU/ml (*P* = 0.0004).

## Discussion

### Samples from HD patients

In the present study, we monitored the time course of changes in the plasma levels of small metabolites during HD and plasma IRI before and after HD in DM and non-DM patients in order to determine whether or not energy failure develops during high-flux HD. The collection of numerous blood samples from HD patients is not appropriate due to their diminished hematogenous function in the kidney; as such, spent dialysate was non-invasively collected without limitation and measured by ^1^H NMR spectroscopy. Quantified levels of small metabolites on dialysate spectrum were converted to their plasma levels based on the established ratios, as mentioned above.

### Metabolites related to energy production

The metabolites illustrated in the Fig 3, and Fig 4 are directly coupled with the main pathway of energy production.

In non-DM patients, lactate, pyruvate and alanine were markedly increased halfway through HD, indicating that their production in the tissue was enhanced during HD. This suggests accelerated glycolysis, which was considered to be stimulated by the meal intake during HD and/or the high glucose concentration of the dialysate. The lowest levels of these 3 metabolites were observed at 30 min (approximately). These metabolites began to increase within the first hour of dialysis. The acceleration of glycolysis seems to have been induced by the glucose in the dialysate, since meals were consumed around the second hour of HD in non-DM patients. Simultaneously, the levels of plasma ketone bodies and acetate remained low throughout the HD. As shown in Fig 4C, the citrate levels of non-DM patients were higher than those of DM patients. Changes in the citrate levels were synchronized with those of lactate, pyruvate and alanine during HD, indicating that the tricarboxylic acid (TCA) cycle was working as it should.

In contrast, in DM patients, the levels of lactate, pyruvate and alanine showed a continuous decrease with time. At the end of HD, the concentrations of these metabolites were below the lower limit of the normal range (as shown in Figs 3A, 3B and 4A), indicating impaired glycolysis. Lactic acidosis is reported to be prevalent in patients with renal failure [18]. However, in the present study, DM patients receiving HD therapy showed hypolactatemia during HD. In addition, low levels of pyruvate during HD in DM patients may cause stagnation of the TCA cycle. In contrast, dramatic increases in the levels of ketone bodies (hyperketonemia) were observed during HD. It has been reported that fatty acids are released from the adipose tissues in DM patients [19]. Oxidation of fatty acid increases the acetate and ketone body levels (Figs 4D and 3D). Such a condition accompanies the elevation of the acetyl-coA levels. Increased acetyl-coA can cause feedback inhibition of the dehydrogenase reaction of pyruvate and accelerate the decarboxylase reaction of pyruvate [20]. These reactions may disrupt the normal stream of energy production thorough the TCA cycle. Inhibition of the TCA cycle decreases the citrate level (Fig 4C), and the TCA cycle stagnates when plasma citrate levels are low [21]. These reactions accompany the activation of the gluconeogenesis pathway through oxaloacetic acid (OAA). As shown in Fig 4A, the steep decrease in the alanine level suggests the recruitment of alanine as a precursor of gluconeogenesis.

### Decreased IRI in DM patients

High-flux membranes have been shown to remove relatively large molecules, such as α_1_-microglobulin (MG) or β_2_-MG [22]. Molecules smaller than α_1_-MG (molecular weight: 33 kDa) can be removed by diffusion through a dialyzer. Insulin (molecular weight: 5.8 kDa) has been reported to be removed by recent HD devices [23] as well as by adsorption by membranes [24]. In the present study, insulin was obviously removed through the membrane (Fig 5B). The plasma levels of IRI in all DM patients decreased, and most of the patients showed insufficient insulin secretion to compensate for the removal during HD. At the end of HD, the IRI level in DM patients was below the lower limit of the normal range. There is a prevailing view in the nephrology community that uremia is an insulin-resistant state [25], so the low IRI in DM patients may indicate poverty of insulin activity.

In contrast, non-DM patients showed a large increase in IRI. The amount of endogenous secretion was considered to be stimulated by the use of dialysate with a high glucose level as well as taking a meal during HD. The increment of IRI was consistent with the behavior of lactate, pyruvate and alanine during HD. Sufficient insulin may enable blood glucose to be transported into tissues as a source of glycolysis. The behaviors of IRI in DM and non-DM patients explained the difference in glycolysis between the two groups.

**Fig 6:**
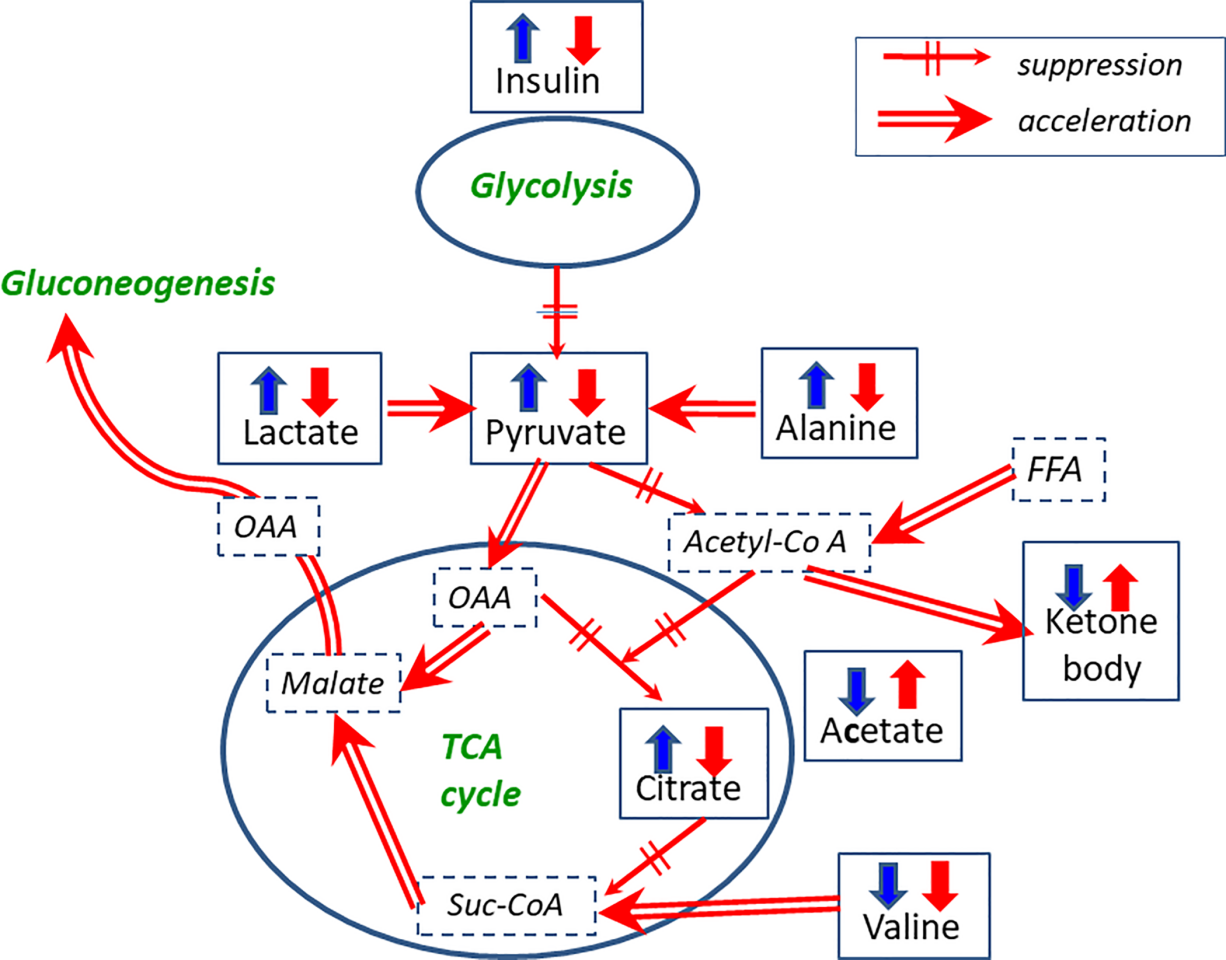
Possible starvation and gluconeogenesis pathways in DM patients. The measured metabolites and insulin are represented in Gothic letters in boxes. Red and blue arrows show the behaviors of metabolite levels in DM and non-DM patients, respectively. Up and down arrows represent high and low values at later times (2–4 h) during HD, respectively. Detailed time course changes of these levels are presented in Figs 3, 4 and 5. Double arrows and dashed single arrows indicate the acceleration and suppression of flow, respectively. OAA, FFA and Suc-CoA represent oxaloacetic acid, free fatty acid and succinyl Co-A, respectively.

Based on these findings, we conclude that the metabolic behaviors of non-DM patients maintained normal homeostasis during HD, while homeostasis during HD in DM patients was disrupted, which represented cell starvation being characterized as ‘hypolactatemia and hyperketonemia’, and also accompanied by gluconeogenetic processes. Potential metabolic pathways in DM patients during HD are illustrated in Fig 6, which is a summarized map of Figs 3, 4 and 5.

### Starvation

There have been no reports of HD-induced starvation in patients undergoing HD using a dialysate with a high concentration of glucose. As depicted in Fig 6, the synchronized profiles of metabolites related to energy production have provided biochemical evidence of cell starvation in DM patients.

Starvation is accompanied by proteolysis in the muscles. Enhanced degradation of valine from muscle protein in the DM patients has been reported [26], and the behavior of valine during HD in DM patients in the present study (Fig 4B) agreed with such results. Some of the catabolized amino acids (mostly alanine) are recruited into the newly emerged gluconeogenesis processes [27]. The recruitment of alanine to the gluconeogenesis pathway well explained the decrement in the alanine levels in Fig 4A. However, HD continuously removes alanine and other amino acids. The subsequent poor supply of such amino acids may thereby inhibit gluconeogenesis. The metabolic consequences of DM patients in HD treatment were suppressed glycolysis, an inhibited TCA cycle, accelerated β-oxidation of fatty acids and insufficient gluconeogenesis. Therefore, such starvation evoked every two days in DM patients with HD therapy may also deplete their glycogen stores and result in unstable glycemic control.

In this way, DM patients receiving HD might develop a serious physiological condition, such as ketoacidosis. As shown in Fig 3D, mild ketosis was occasionally observed in DM patients, although this ketosis remained at the physiological level. However, when insulin is deficient, ketosis can progress to ketoacidosis (3–5 mM) [19].

In the present study, the insulin depletion at the end of HD led to starvation of a catabolic state. We therefore concluded that insulin therapy during HD was indispensable for DM patients with insufficient insulin secretion. Exogenous insulin or anti-diabetic agents may need to be administered early in the HD session in order to ensure good glycemic control. Of note, insulin has anabolic roles in the muscle and whole-body metabolism [28]. If intradialytic insulin is administered, DM patients should be encouraged to take meals carefully in order to maintain glycemic and blood pressure control. Furthermore, under the anabolic condition achieved, intradialytic amino acid supplementation would be efficient for nutrition to compensate the removal by HD. These treatments may prevent patients from progressing to sarcopenia. It may be essential to maintain glucose homeostasis even during HD treatment.

Hemodialysis itself is considered a catabolic procedure because of the associated enhanced inflammation, removal of amino acids and other metabolic abnormalities [29], and HD patients are prone to be in a catabolic state. The present study revealed novel metabolic evidence of cell starvation in DM patients receiving HD that risks inducing further profound catabolism via mechanisms different from those previously recognized. While insulin removal through HD has been recently reported, its immediate and fatal effects on the whole metabolism of DM patients have not been noted before.

### Study limitations

In the present study, DM and non-DM patients were not matched regarding the presence or absence of a meal intake. We assumed that the high glucose concentration (150 mg/dl) in the dialysate had an additive effect with meal-taking on the glucose metabolism of non-DM patients, with little effect noted in DM patients.

## Conclusion

In the present study, we presented biochemical evidence of cell starvation, observed as hypolactatemia and hyperketonemia, in HD-DM patients even when using a dialysate with a high glucose concentration. Insulin was depleted by high-performance HD, resulting in cell starvation in DM patients. Recurrent starvation episodes under conditions of insulin deprivation may predispose patients to sarcopenia through proteolysis and lipolysis. To avoid the progression of sarcopenia, homeostasis of the glucose metabolism should be maintained to control the plasma concentrations of insulin. To this end, the intradialytic catabolic state of DM patients might be shifted to an anabolic state by the administration of exogenous insulin and amino acid supplementation during HD sessions. Further studies will be necessary to establish detail remedies tailored to DM patient.

## Supporting information

S1 Table Shows patients’ numbers, kinds, doses of antidiabetic medications, and the comments. (DOCX)
