# Supplementary material for: Biochemical evidence of cell starvation in diabetic hemodialysis patients
Source: PLoS One. 2018 Sep 27;13(9):e0204406. doi: 10.1371/journal.pone.0204406 (PMC6160080; doi:10.1371/journal.pone.0204406)
Supplement: S1 Table — (DOCX) [file pone.0204406.s001.docx]

S1 Table. Antidiabetic medications in 10 DM patients.

| Patient No |  | E |  | comments |
| --- | --- | --- | --- | --- |
| 1 | intermediate acting insulin | 10 - 0 - 0 |  |  |
| 2 | rapid acting insulin | 2 - 2 - 2 |  |  |
|  | long acting insulin | 0 - 0 - 6 |  |  |
| 3 | intermediate acting insulin | 12 - 0 - 0 |  |  |
| 4 | rapid acting insulin | 6 - 0 - 6 |  |  |
|  | intermediate acting insulin | 0 - 4 - 4 |  |  |
| 5 | piogliyazone　1T | 15mg after breakfast | to increase sensitivity to insulin | |
| 6 | Tenegliptin 2T | 20mg after breakfast | DPP-4 inhibitor | |
| 7, 8, 9,10 | no medication |  |  |  |

Patient’s no. 2 and 4 had no insulin injections during HD.
